# Veratrium Viride in Puerperal Convulsions

**Published:** 1882-10-20

**Authors:** R. F. McConnell

**Affiliations:** Alabama


### Southern Medical Record:

EDITORS:

T. S. POWELL, M.D. W. T. GOLDSMITH, M.D. R. C. WORD, M.D.

*R. C. WORD, M.D., Managing Editor.*

☞All Communications and Letters on Business connected with the RECORD must be addressed to the Managing Editor.

VOL. XII. ATLANTA, GA., OCT. 20, 1882. NO. 10.

### ORIGINAL AND SELECTED ARTICLES.

### VERATRIUM VIRIDE IN PUERPERAL CONVULSIONS

BY R. F. MCCONNELL, M.D., OF ALA.

I desire to report some obstetrical cases and their treatment.

CASE 1. March 13th, 1882. Called to see Mrs. D., æt. 20. Primipara; robust build; weight, perhaps 150 ℔s.; attendants said she had been healthy. On my arrival found patient had been delivered about two hours previously of a fine daughter, weighing about 10 ℔s. She was suffering with severe headache and pain in right inguinal region: the latter she complained of while being delivered of child. I delivered her of placenta in twenty minutes after my arrival. At this time headache was increasing rapidly, and I gave her 20 grs. brom. pot. without any special benefit and repeated dose in half an hour. She had a convulsion in ten minutes (an hour after my arrival) and, unfortunately, I had no arterial sedative, and did not wish to perform venesection. Therefore I resorted to the following remedies, viz.:

℞. Bromide pot. . . . . . . . . . . . . . . . . . . . . . . . . . . . . . . . grs. 80

Hyd. chloral . . . . . . . . . . . . . . . . . . . . . . . . . . . . . . . . " 40

Aqua . . . . . . . . . . . . . . . . . . . . . . . . . . . . . . . . . . . . ℥ 1

Sig. Tablespoonsful every ½ hour.

Also, tr. aconite, gtts. ii, every ½ hour.

This treatment, with an occasional dose of Hoffman's Anodyne and a sinapismto nape of neck, was continued until 4 p. m. At this time I received some veratrum viride. She had been having severe convulsions every hour till 12 a. m. when they ceased until 2 p. m. Then they returned with increased severity and frequency, coming on at intervals of an hour or less. Pulse, 122 per minute, with contracted pupils, etc. I now added following to former treatment, viz.:

℞. Tr. veratrum viride....................... gtts. 4

Hyd. chloral............................. grs. 40

Aqua...................................... ℥ 1

Sig. Tablespoonful every ½ hour.

At 4½ o'clock patient had three convulsions at short intervals; also at 5 o'clock p. m. and continued until 6 o'clock, when they ceased.

This treatment was continued during the night. Patient would rest well from half an hour to an hour and then awake perfectly delirious, refusing to take medicine and attempting to get up and to fight any who opposed her. It required from three to four persons to hold her in bed while aroused.

March 14, 5 a. m. Patient delirious all day, with a gradual decrease in circulation. Pulse 116, respiration 14. She would rest from half an hour to an hour and then show signs of great excitement. I continued treatment during night, with signs of improvement until

March 15, 5 a. m., when she awoke and appeared to be sensible, making some inquiry about the condition of her tongue, which had been badly bitten during convulsions.

March 16, 5 a. m. Pulse 108. I ordered verat. mxt. given in tablespoonful doses every hour. Patient continued about in this condition, with an occasional increase in the frequency of the pulse.

March 17, 5 a. m. Pulse, 102 to 108, with an occasional decrease to as low as 90. At noon pulse rose to 122. Ordered verat. mxt. every half hour until circulation was reduced.

March 18, 9 a. m. Pulse 82, with good symptoms of improvement. During day I again ordered veratrum viride and bromide mxt. every hour and she continued to improve until about noon of

March 19, when fever appeared. Pulse 120. Patient complained of left mammæ, in which secretion of milk was checked, also of sick stomach. Her bowels had not moved since the 15th, at which time a purgative had been given. I now gave her a dose of mercury and ordered following given in connection with previous treatment, viz.:

℞. Tr. aconite (rad.) . . . . . . . . . . . . . . . . . . . . . . . . . ʒss

Sweet spts. nitre . . . . . . . . . . . . . . . . . . . . . . . . . ℥j

Tr. lavender comp. . . . . . . . . . . . . . . . . . . . . . . . . gtts. xvi

Aqua . . . . . . . . . . . . . . . . . . . . . . . . . . . . . . . . . ℥ji

Sig. Teaspoonful every 2 hours.

This treatment was continued until fever subsided, and at 9 a.m. 20th inst. she showed marked signs of improvement, and continued so, with slight evening exacerbations of fever, until March 26, when I dismissed case. I was compelled to exercise great caution in decrease of veratrium mxt., which was used up to present date. Whenever veratrum was much reduced patient would have paroxysms of fever and cerebral disturbance.

CASE 2. April 29, 1882. At 10 a. m. called to see Mrs. B., æt. 20, seven months advanced in pregnancy. Primipara; robust build; would weigh about 165 pounds. Attendants say she had previously exposed herself, and had had some suppression of urine, but had not complained much until the night previous. From symptoms I decided that she had some uræmic poisoning; had been troubled with headache and suppression of urine for a month; had a convulsion at 5 a. m. and up to time of my arrival at 10 a. m. had had about 15 convulsions. Pulse 108; breathing frequent and stertorous; examination showed first stage of labor begun—os uteri thin and slightly dilated.

℞ Bromide pot . . . . . . . . . . . . . . . . . . . . . . . . . . . . . grs. xx
Hyd. chloral . . . . . . . . . . . . . . . . . . . . . . . . . . . . " xv
Every ½ hour.

Also the following:

℞ Veratrum viride . . . . . . . . . . . . . . . . . . . . . . . . . . . gtts ji

Every ½ hour.

Convulsions and pain appeared synchronously every 15 minutes, with very slow advancement of labor and the same degree of severity, until 6 p. m. At this time convulsions began to increase. I had given two ʒj doses of ergot to increase labor. Convulsions continued to increase in severity, with no advancement of labor, until 7½ p. m. Pulse now 160 and irregular; slow and stertorous breathing; comatose; frothing at mouth; vertex presenting at inferior outlet. Labor had been thus for two hours. I could not stimulate labor by any means. Having no forceps, I performed craniotomy and delivered her. Treatment was continued and she had but two convulsions after delivery. Patient remained in a comatose condition during night.

April 30, 7 a. m. Pulse 108; at 3 p. m. pulse 122. Ordered veratrum viride, gtts ji, every two hours until 12 p. m.; then every three hours till 9 a. m.

May 1st. Pulse 76. Ordered veratrum given whenever circulation ran over 100.

May 2d, 8 a. m. Pulse 114. Ordered veratrum every two hours, as before, until circulation was reduced to 80, and then given whenever it rose. Patient became quiet at 10 p. m. and continued so until

May 3, 7 a. m. At this time appeared to be approaching sanity.

May 4, 6 a. m. Patient had become perfectly sane. She had had about 51 convulsions.

I have made this report to show the great benefit derived from verat. viride in the treatment of puerperal convulsions.

